# Validation for use in Colombia of the EORTC QLQ–THY34 questionnaire to measure quality of life in patients with thyroid cancer

**DOI:** 10.1007/s11136-026-04392-9

**Published:** 2026-09-21

**Authors:** Laura Johanna Piña-Jaramillo, Diana Isabel Cuéllar-Rivera, Andrés Arturo Cuéllar-Cuéllar, Ricardo Sánchez-Pedraza

**Affiliations:** 1 Departamento de Epidemiologia Clínica y Bioestadística, Facultad de Medicina, Universidad Nacional de Colombia, Bogotá, D.C Colombia; 2 Subdirección de Investigaciones, Fundación Cardioinfantil-Instituto de Cardiología, Bogotá, D.C Colombia; 3 Grupo de Investigación Clínica y Epidemiológica del Cáncer, Grupo Área de Investigaciones, Instituto Nacional de Cancerología, Bogotá, D.C Colombia; 4 Unidad de endocrinología oncológica, Grupo Área Unidades Médicas, Instituto Nacional de Cancerología, Bogotá, D.C Colombia; 5 Instituto de Investigaciones Clínicas, Facultad de Medicina, Universidad Nacional de Colombia, Bogotá, D.C Colombia

**Keywords:** Thyroid cancer, Quality of life, Questionnaire validation, Psychometric validation, Patient-reported outcomes, Rasch analysis

## Abstract

**Purpose:**

Although survival after thyroid cancer is favorable, many patients experience persistent symptoms and concerns that affect health-related quality of life (HRQoL). This study evaluated the measurement properties of the Colombian Spanish EORTC QLQ-THY34 in patients with thyroid cancer.

**Methods:**

500 adults treated at a national oncology referral center were included. Psychometric validation was conducted using Classical Test Theory (CTT) and Rasch analysis. CTT evaluated internal consistency; structural, convergent, and divergent validity; test–retest reliability, and responsiveness. Rasch analysis examined dimensionality, item and person fit, reliability, differential item functioning (DIF), and the relationship between item difficulty and patient responses using the person–item map.

**Results:**

The empirical four-domain model: (Symptoms and Impact, Cancer-related Concerns, Oropharyngeal Alterations, and Lack of Social Support), fitted substantially better than the original 17-scale model, although RMSEA and SRMR remained above conventional thresholds. Internal consistency was adequate across domains, and Test-retest reliability was good for three domains and moderate in one. Responsiveness analyses showed statistically significant changes over time in the Symptoms and Impact and Cancer-Related Concerns domains. Rasch analysis indicated acceptable fit for most items, although some exhibited DIF, and suggested usefulness in patients with greater clinical complexity.

**Conclusion:**

The Colombian Spanish EORTC QLQ-THY34 showed generally adequate measurement properties, supporting its use for assessing health-related quality of life in patients with thyroid cancer in Colombia. The empirically derived four-domain structure should be considered preliminary and requires replication in independent and more clinically diverse samples; meanwhile, the original EORTC scoring system should remain the standard for cross-study comparisons.

**Supplementary Information:**

The online version contains supplementary material available at https://doi.org/10.1007/s11136-026-04392-9.

## Abstract (plain language summary)

Thyroid cancer often has a favorable prognosis, but many patients experience persistent symptoms and concerns that affect daily life. We evaluated the Colombian Spanish version of the EORTC QLQ-THY34, a questionnaire designed to assess health-related quality of life in people with thyroid cancer. The study included 500 adults treated at a national cancer referral center. The questionnaire was assessed to determine whether it met established psychometric requirements. The results showed that the questionnaire can be grouped into four main areas: Symptoms and Impact, Cancer-related Concerns, Oropharyngeal Alterations, and Lack of Social Support. It was found to be consistent and stable over time, and capable of detecting changes after surgical reintervention. Additional analyses showed that most items performed well and that the questionnaire may be particularly useful in patients with more complex clinical conditions. The original EORTC scoring system should continue to be used for comparisons across studies while the alternative structure is evaluated in independent and clinically diverse samples.

**Supplementary Information:**

The online version contains supplementary material available at https://doi.org/10.1007/s11136-026-04392-9.

## Introduction

Thyroid cancer (TC), the most common endocrine malignancy, has increased in incidence while survival remains high for most patients. Consequently, HRQoL is an important outcome [1]. In Colombia, TC ranks fifth in cancer incidence in both sexes [2].

Patient-reported outcome measures (PROMs) capture patients’ perceptions of the disease and treatment [3]. Disease-specific PROMs may be particularly relevant in TC because they address symptoms and concerns not fully covered by generic instruments [4, 5].

The EORTC QLQ-THY34 is a TC–specific module developed by the European Organisation for Research and Treatment of Cancer [6]. Although internationally evaluated, its measurement properties should be examined in the cultural context in which it will be used.

This study therefore evaluated the measurement properties of the Colombian Spanish EORTC QLQ-THY34 [6], using CTT [7] and Rasch model analyses [8].

## Materials and methods

This psychometric validation study followed a previously completed cross-cultural adaptation of the EORTC QLQ-THY34 for Colombia [9], conducted according to EORTC Quality of Life Group procedures [10]. Briefly, three subject-matter experts who were native speakers of Colombian Spanish reviewed the Spanish version for Spain and proposed wording changes to 7 of the 34 items to improve semantic clarity without changing the intended constructs. Subsequently, a pilot test was carried out through semi-structured cognitive interviews with 15 patients with varied sociodemographic characteristics. Participants reported no items as confusing, offensive, or difficult to understand, and no further changes were required [9].

### Instrument

The EORTC QLQ-THY34 contains 34 items rated on a four-point response scale and is scored on a standardized 0–100 scale, with higher scores indicating greater symptom burden or HRQoL impairment [6].

### Participants

Eligible participants were Spanish speaking Colombian adults (≥ 18 years) with histopathologically confirmed thyroid cancer of any histological type. Patients were excluded if they had a second primary malignancy, except non-melanoma skin cancer, documented sensory or cognitive impairment that could hinder comprehension or questionnaire completion; or if they declined participation. Consecutive sampling was used. Eligible patients attending the Endocrinology Service were approached in person or by telephone and provided written or verbal informed consent. The EORTC QLQ-C30 and EORTC QLQ-THY34 were administered in accordance with EORTC recommendations [16].

### Sample size

Sample size was determined separately for each measurement property. A minimum of 78 participants was estimated for convergent validity [11], 96 for internal consistency [12], 81 for test–retest reliability [13], and 42 for responsiveness [17]. For structural validity, a total sample of 500 participants was planned to permit random division into two independent subsamples of 250 participants, one for exploratory factor analysis and the other for confirmatory factor analysis. This split-sample approach was intended to reduce capitalization on chance and to allow independent evaluation of the empirically derived factor structure [14, 15]. For Rasch analysis, a minimum sample of 400 participants was considered adequate in accordance with EORTC recommendations [16]. The assumptions, software, and detailed sample size calculations for each measurement property are provided in Supplementary Material 1 [11–17].

### Measurement

Face-to-face assessments were completed before the scheduled clinical consultation, whereas telephone assessments were arranged according to participants’ availability. For test–retest reliability, a non-probability subsample completed the baseline assessment face to face before their scheduled clinical consultation and was contacted by telephone 4–7 days later to complete the retest. Participants were eligible for retesting when no medical or surgical interventions were planned during this interval to reduce the likelihood of clinically important change between measurements.

For the responsiveness analysis, patients undergoing surgical reintervention completed two additional assessments at approximately 3 and 6 months postoperatively. All questionnaires were administered as structured interviews by trained personnel experienced with the instruments. Responses were entered directly into electronic case report forms developed in REDCap^®^ [18].

### Statistical analysis

Continuous variables were summarized using means or medians, depending on their distribution, along with their corresponding measures of dispersion (standard deviation [SD] or interquartile range [IQR]). Categorical variables were reported as absolute and relative frequencies. Analyses were performed using R software [19]. EORTC QLQ-C30 and EORTC QLQ-THY34 scores were calculated according to the EORTC scoring manual [20].

### Validation under classical test theory

Structural validity: Although the EORTC QLQ-THY34 has an established structure, EFA was included because this was its first psychometric evaluation in a Latin American population and the performance of the original structure could not be assumed to generalize across cultural contexts. To reduce the risk of capitalizing on chance, a split-sample design was used: exploratory factor analysis (EFA) was conducted in one randomly selected subsample, and confirmatory factor analysis (CFA) was performed in the independent subsample to compare the original EORTC structure with the empirically derived model [21]. Because the items were ordinal, EFA was based on a polychoric correlation matrix. Factorability was assessed using Bartlett’s test [22] and the Kaiser–Meyer–Olkin statistic [23]. Factor number was informed by parallel analysis, scree plot, and the Kaiser criterion [24]. Weighted least-squares (WLS) extraction and both orthogonal and oblique rotations were evaluated, considering statistical adequacy, simple structure, and clinical interpretability [25, 26]. CFA used the Weighted Least Squares Mean and Variance adjusted (WLSMV) estimator. Model fit was assessed using standard goodness-of-fit indices, including Root Mean Square Error of Approximation (RMSEA ≤ 0.08), Root Mean Square Residual (RMR close to 0), Standardized Root Mean Square Residual (SRMR ≤ 0.08), Adjusted Goodness-of-Fit Index (AGFI ≥ 0.90), Comparative Fit Index (CFI ≥ 0.95), Tucker–Lewis Index (TLI ≥ 0.95) and Normed Fit Index (NFI ≥ 0.90), to assess both the original EORTC model and the structure derived from the EFA [27, 28]. The CFA subsample (*n* = 250) was subsequently used to estimate internal consistency and to assess convergent and divergent validity, thereby preserving independence from the subsample used to derive the empirical factor structure. Internal consistency was estimated with Cronbach’s alpha [29, 30] and McDonald’s omega, and the greatest lower bound (GLB), including item-deletion [31–33]. Convergent and divergent validity were examined through Spearman’s correlation between the empirical domains and EORTC QLQ-C30 [11, 32]. Test–retest reliability was evaluated with Lin’s concordance correlation coefficient (CCC) [13].

Responsiveness was explored by comparing scores at baseline and approximately 3 and 6 months after surgical reintervention. Overall change was tested with Friedman test, followed by Bonferroni-adjusted pairwise Wilcoxon signed-rank tests [34, 35].

A random subsample of 400 participants from the baseline assessment was selected for Rasch analysis using a fixed seed of 123 to ensure reproducibility. Rasch analysis was conducted using the Rating Scale Model and included all 34 items as a complementary evaluation of the complete item bank along an overarching continuum of thyroid-cancer-specific symptom, problem, and concern burden affecting HRQoL. This analysis was not intended to support the calculation of a total score or to replace the multidomain EORTC scoring structure. The Rasch analysis evaluated overall model fit, item and person fit, item difficulty, person–item targeting, person and item reliability and separation, category functioning, local item dependence, differential item functioning, and unidimensionality based on principal component analysis of standardized residuals [36–39].

All analyses were performed using R and Winsteps^®^ software. Additional methodological details are provided in the Supplementary Material 2.

## Results

### Sample characteristics

Between September 18, 2023, and July 10, 2024, 523 participants were enrolled; 23 with a secondary primary malignancy were excluded. The participant recruitment and analysis flow is presented in Supplementary Material 3. Follow-up for the responsiveness subgroup ended in February 2025. Participant characteristics are shown in Table 1.

**Table 1:** Sociodemographic and clinical characteristics of the study population

Age, mean (SD)		52,28	(14,2)
Sex, n (%)	Female	425	(85)
Cultural region, n (%)	Cundiboyacense regionᵃ	192	(38,4)
	Cachaca regionᵇ	122	(24,4)
	Tolimense regionᶜ	70	(14)
	Santandereana regionᵈ	28	(5,6)
	Paisa regionᵉ	27	(5,4)
	Caribbean regionᶠ	24	(4,8)
	Other cultural regions ᵍ	37	(7.4)
Years of education, median (IQR^l^)		11	(5–13)
	No formal education	11	(2.2)
	Incomplete primary	42	(8.4)
	Completed primary	84	(16.8)
	Incomplete secondary	60	(12)
	Completed secondary	123	(24.6)
	Higher education	145	(29)
	Postgraduate	35	(7)
Marital status, n (%)	With partnerᵐ	273	(54,6)
	Without partnerⁿ	227	(45,4)
Employment status, n (%)	Not employedᵒ	272	(54,4)
	Employedᵖ	215	(43)
	Student	13	(2,6)
Socioeconomic status, n (%)	1–3	467	(93,4)
	4–6	33	(6,6)
ECOG performance status, n (%)	0	458	(91,6)
	1	37	(7,4)
	2	4	(0,8)
	3	1	(0,2)
Charlson comorbidity index, n (%)	0	49	(9,8)
	1–2	78	(15,6)
	3–4	213	(42,6)
	> 5	160	(32)
Histological type, n (%)	Papillary	477	(95,4)
	Follicular	13	(2,6)
	Medullary	9	(1,8)
	Anaplastic	1	(0,2)
Stage, n (%)	I	325	(65)
	II	115	(23)
	III	15	(3)
	IVA	1	(0,2)
	IVB	20	(4,0)
	Unknown	24	(4,8)
Surgery, n (%)	Total thyroidectomy	483	(96,6)
	Partial thyroidectomy	15	(3)
	No surgery	2	(0.4)
Neck dissection, n (%)	Central	209	(41,8)
	Central + unilateral	152	(30,4)
	Central + bilateral	48	(9,6)
	Unilateral	24	(4,8)
	Bilateral	14	(2,8)
	No dissection	45	(9)
	Missing	8	(1,6)
Radiotherapy, n (%)		22	(4,4)
Radioiodine therapy, n (%)		379	(75,8)
Targeted therapy, n (%)		20	(4,0)
Palliative care, n (%)		25	(5,0)

Note: ^a^Cundinamarca and Boyacá; ^b^Bogotá; ^c^Huila and Tolima; ^d^Santander and Norte de Santander; ^e^Antioquia, Caldas, Quindío, and Risaralda; ^f^Atlántico, Bolívar, Cesar, Córdoba, La Guajira, Magdalena, San Andrés and Providencia, and Sucre; ^g^Amazon region: Amazonas, Caquetá, Guainía, Guaviare, Putumayo, Vaupés, and Vichada; Llanera region: Arauca, Casanare, and Meta; Valluna: Valle del Cauca and Cauca; Serrana: Nariño; IQR = interquartile range; ^m^married or cohabiting; ^n^single, separated, or widowed; ^o^ unemployed, retired, or homemaker; ^p^ employed or self-employed

### Item characteristics

**Fig. 1:**
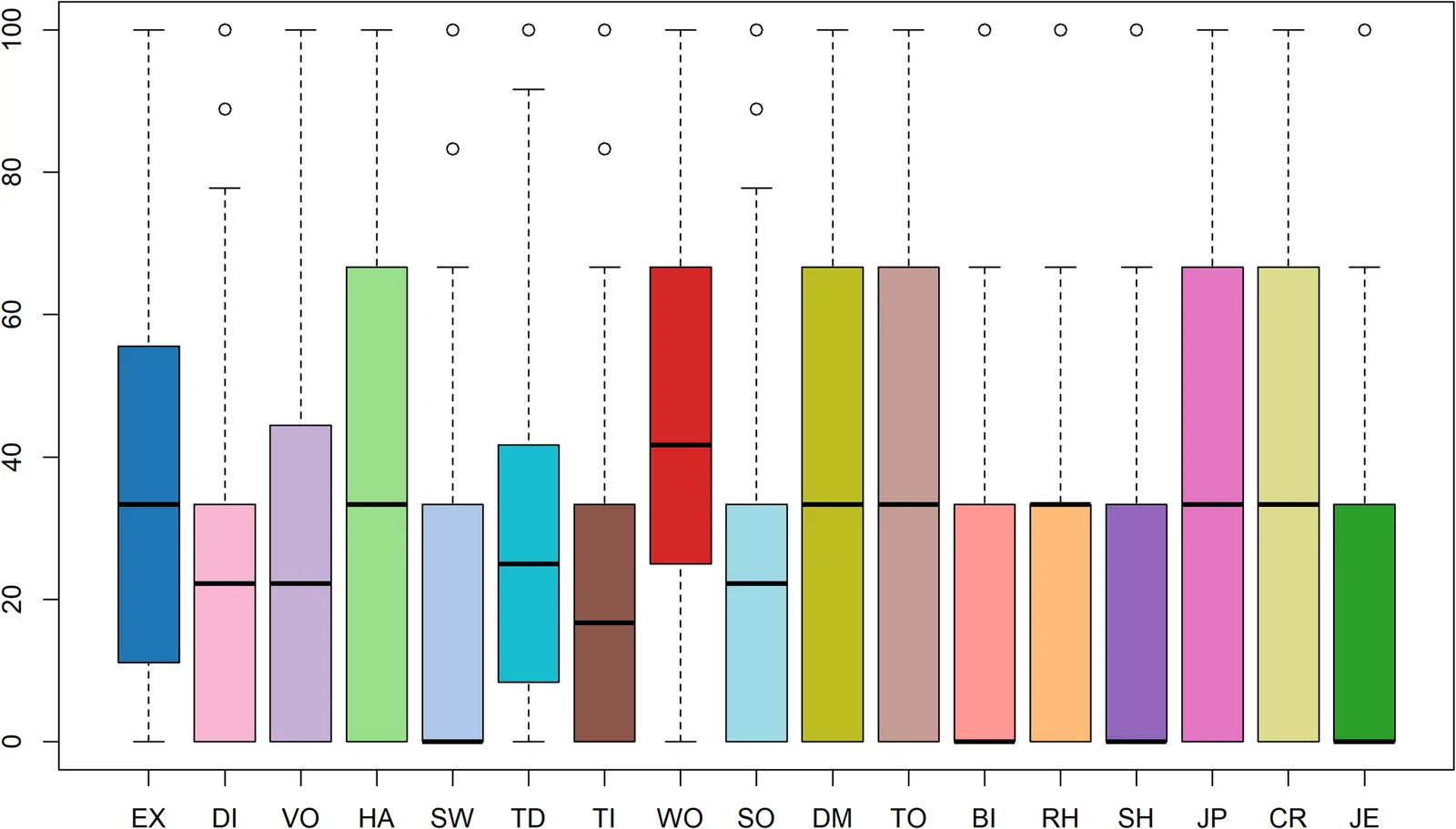
Distribution of the original EORTC QLQ-THY34 scale in the study population. Boxplots were generated in R. EX: Exhaustion; DI: Discomfort in the Head and Neck; VO: Voice; HA: Hair Problems; SW: Swallowing; TD: Treatment- and Disease-Related Worry; TI: Tingling or Numbness; WO: Worry About Important Others; SO: Lacking Social Support; DM: Dry Mouth; TO: Altered Temperature Tolerance; BI: Body Image; RH: Rapid Heartbeat; SH: Shoulder Functioning; JP: Joint Pain; CR: Cramps; JE: Impact on Job or Education

All participants completed both questionnaires with no missing item responses. The highest original EORTC QLQ-THY43 scale scores were observed for *Worry about Important Others* and *Exhaustion*, with median values of 41.67 (IQR: 25–66.67) and 33.33 (IQR: 11.11–55.56), respectively. Figure 1 illustrates the distribution of scale scores across the study population.

### Classical test theory validation

#### Exploratory factor analysis

The correlation matrix was suitable for factor analysis (Bartlett’s test: χ² = 6841.013, df = 561, *p* < 0.01; KMO = 0.759). Parallel analysis supported four factors, consistent with the Kaiser criterion and scree-plot inspection (Supplementary Material 4); together they explained 52% of the variance. Oblique rotation produces the most clinically interpretable solution. Most items loaded ≥ 0.40, except item 48 (loading 0.36; uniqueness 0.83). The factors were named Symptoms and Impact (SI), Lack of Social Support (SO), Cancer-Related Concerns (CRC), and Oropharyngeal Alterations (OA). Item 50 and 52 cross-loaded and were assigned to CRC on clinical grounds. Supplementary Material 5 presents the item stems, the mapping to the original EORTC scales and the empirically derived domains, as well as the factor loadings and uniqueness values obtained from the exploratory factor analysis.

### Confirmatory factor analysis

CFA compared the original EORTC structure with the EFA-derived model. The empirical model met conventional thresholds for most indices and showed substantially better fit than the original model, which met none of the prespecified criteria. Nevertheless, RMSEA (0.085) and SRMR (0.097) remained above conventional cutoffs, indicating that fit was improved but not uniformly optimal (Table 2).

**Fig. 2:**
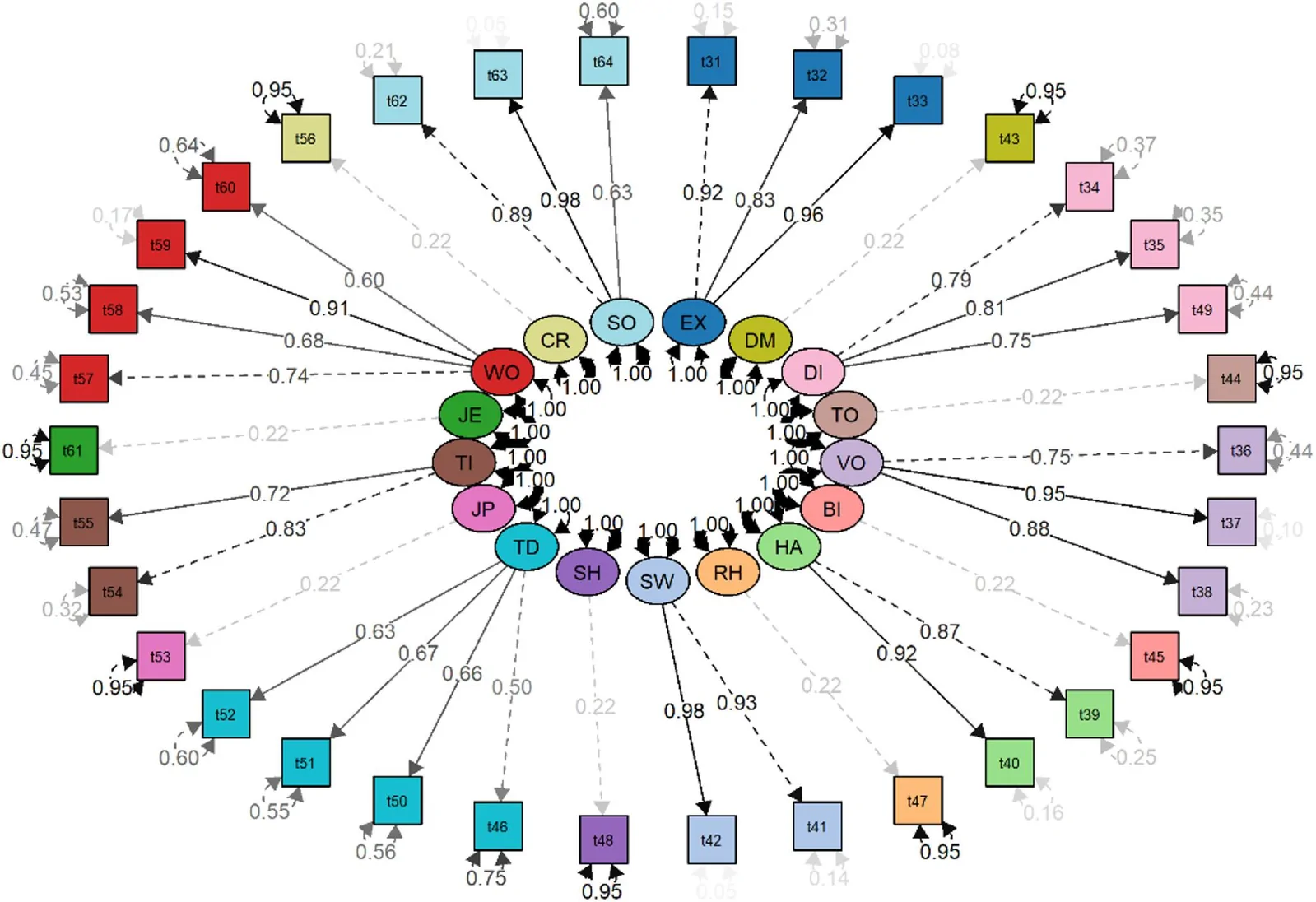
Path diagram of the theoretical model, generated using R software. EX, Exhaustion; DI, Discomfort in the Head and Neck; VO, Voice; HA, Hair Problems; SW, Swallowing; TD, Treatment- and Disease-Related Worry; TI, Tingling or Numbness; WO, Worry About Important Others; SO, Lacking Social Support; DM, Dry Mouth; TO, Altered Temperature Tolerance; BI, Body Image; RH, Rapid Heartbeat; SH, Shoulder Functioning; JP, Joint Pain; CR, Cramps; JE, Impact on Job or Education

**Fig. 3:**
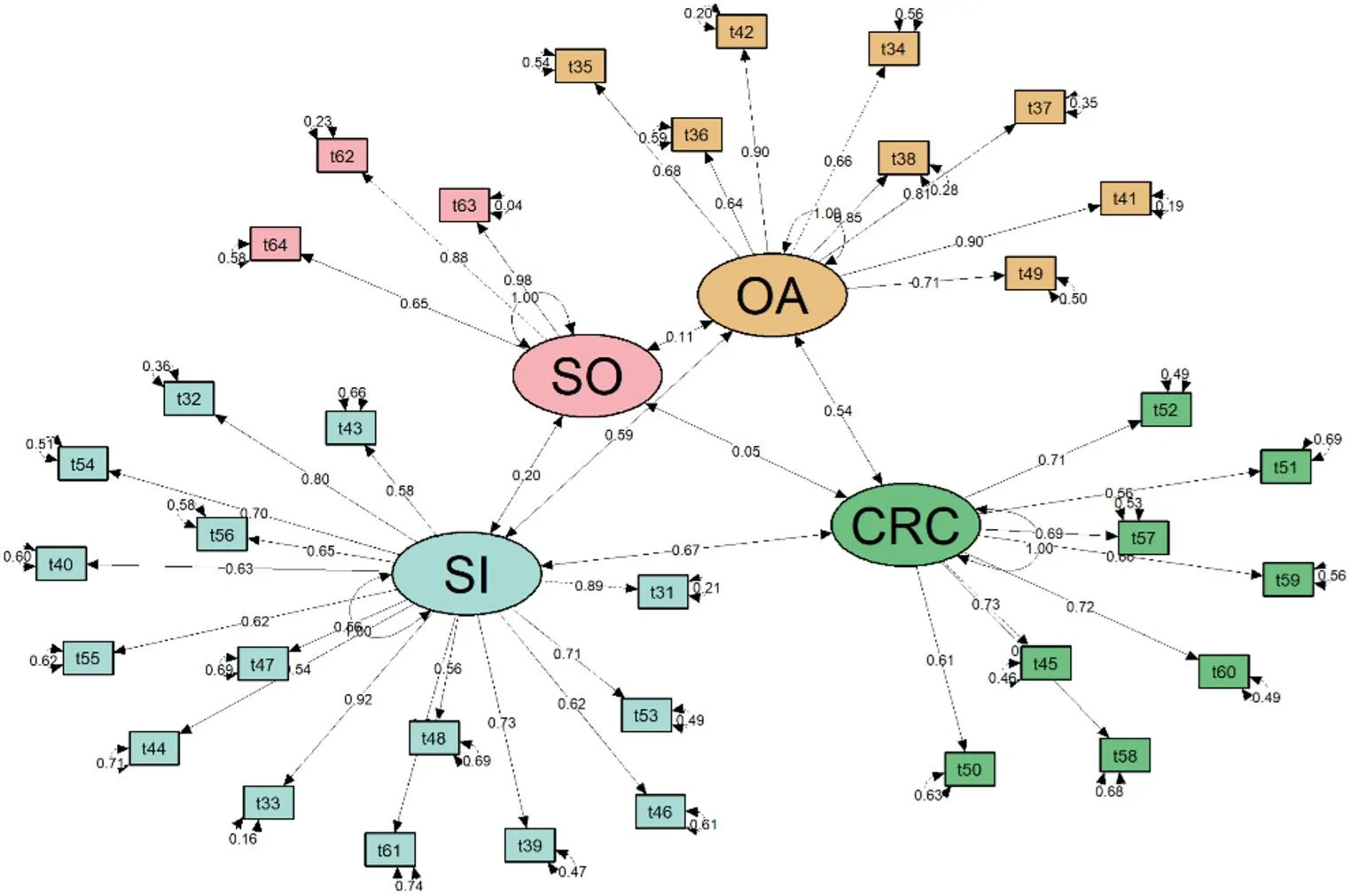
Path diagram of the empirical model, generated using R software. SI: Symptoms and Impact, SO: Lack of Social Support, CRC: Cancer-Related Concerns, and OA: Oropharyngeal Alterations

Path diagrams for both models are shown in Figs. 2 and 3. The empirical structure provided a more parsimonious representation of the present data. Study-specific domain scores derived from this structure were used for exploratory analyses of construct validity, internal consistency, test-retest reliability, and responsiveness (Supplementary Material 6). These scores are not proposed as a replacement for the official EORTC scoring system and should not be used for cross-study comparison without independent replication.

**Table 2:** Goodness-of-Fit Indices for Each Model

Model	X^2^/df^a^	GFI^b^	RMSEA^c^	RMR^d^	SRMR^e^	AGFI^f^	CFI^g^	TLI^h^	NFI^i^	IFI^j^	RFI^k^
EORTC	23.267	0.644	0.299	0.270	0.292	0.552	0.553	0.525	0.543	0.554	0.514
Empirical	2.820	0.957	0.085	0.089	0.097	0.946	0.964	0.961	0.945	0.964	0.941

Note: ^a^Chi−square degrees−of−freedom ratio; ^b^Goodness−of−Fit Index; ^c^Root Mean Square Error of Approximation; ^d^Root Mean Square Residual; ^e^Standardized Root Mean Square Residual; ^f^Adjusted Goodness−of−Fit Index; ^g^Comparative Fit Index; ^h^ Tucker–Lewis Index; ^i^ Normed Fit Index; ^j^ Incremental Fit Index; ^k^ Relative Fit Index

### Internal consistency

Cron^b^ach’s alpha, McDonald’s omega, and the Greatest Lower Bound (GLB) coefficients were estimated. For the complete item set, the coefficients were 0.91, 0.93, and 0.99, respectively. When each item was removed individually, Cronbach’s alpha and McDonald’s omega remained between 0.89 and 0.99, while the GLB coefficient remained stable at 0.99. When internal consistency was examined by domain according to the confirmatory factor analysis, the item-deletion analysis within each domain showed improved Cronbach’s alpha and McDonald’s omega values upon exclusion of item 64, reaching 0.93 and 0.90, respectively (Supplementary appendix 7).

### Floor and ceiling effects

A substantial floor effect was observed for SO (34.8%), indicating that more than one-third of participants selected the lowest possible lack-of-support score. Floor effects were 2.0% for SI and CRC and 12.4% for OA. Ceiling effects were negligible (SI 0.0%, SO 0.4%, CRC 0.0%, OA 0.0%) [40].

### Convergent and divergent Validity

Correlations with the EORTC QLQ-C30 scales showed a pattern broadly consistent with conceptual expectations. SI correlated strongly with Fatigue (*r* = 0.750), Pain (*r* = 0.656), and Global Health Status (*r* = -0.646), and moderately with several other functioning and symptom scales. CRC and OA generally showed correlations of approximately 0.30–0.48 with related scales. SO correlations were uniformly weak. This pattern provides preliminary convergent and discriminant evidence, although the weak SO correlations may reflect both conceptual distinctness from the core questionnaire and restricted score variability caused by the substantial SO floor effect (Supplementary appendix 8).

### Responsiveness

Forty-six patients completed all three assessments and were included in the responsiveness analysis. The second assessment occurred at a median of 92 days (IQR 91–92) and the third at 184.5 days (IQR 183–186). SI and CRC scores changed significantly over time (Friedman *p* = 0.02 and *p* < 0.01, respectively). Bonferroni-adjusted comparisons showed differences between baseline and both postoperative assessments for these domains. OA and SO scores varied descriptively, but their overall changes were not statistically significant (Table 3).

**Table 3:** Responsiveness analysis: domain scores across time points and friedman test results

Domain	Median (IQR) - M0	Median (IQR) – M2	Median (IQR) – M3	Friedman Test	Pos hoc comparisons^a^
SI	40(24.44-55)	27.78(20.56–43.89)	34.44(20 -44.44)	χ2 = 8.0; df = 2; *p* = 0.02	M0–M2: *p* = 0.01
					M0–M3: *p* = 0.04
					M2–M3: *p* = 1
CRC	43.75(30.21–66.67)	37.5(21.88–54.17)	39.58(21.88–53.12)	χ² = 11.47; df = 2; *p* < 0.01	M0–M2: *p* = 0.02
					M0–M3: *p* = 0.01
					M2–M3: *p* = 1
SO	27.78(16.67–44.44)	22.22(11.11–33.33)	22.22(6.67–41.67)	χ2 = 2.53; df = 2; *p* = 0.28	-
OA	22.22(0-33.33)	16.67(0-33.33)	27.78(0-33.33)	χ2 =2.33; df = 2; *p* = 0.31	-

Note: M0 = preoperative assessment; M2 = second measurement; M3 = third measurement; IQR = interquartile range; df = degrees of freedom; ᵃ Wilcoxon signed-rank test with Bonferroni correction

### Test–retest reliability

Eighty-one patients completed the retest after a median of 6 days (IQR 4–7). CCCs indicated good test-retest concordance for SI (0.872), CRC (0.806), and OA (0.867), and moderate concordance for SO (0.666) (Table 4).

**Table 4:** Test–retest reliability:

Domain	CCC	95% CI	Cb
SI	0.872	0.809–0.915	0.983
SO	0.666	0.526–0.771	0.995
CRC	0.806	0.714–0.870	0.996
OA	0.867	0.803–0.911	0.986

Note: CCC, Lin’s concordance correlation coefficient; CI, confidence interval; C_b, bias correction factor; SI, Symptoms and Impact; SO, Social Support; CRC, Cancer-Related Concerns; OA, Oropharyngeal Alterations

### Rasch model validation

#### Unidimensionality and local independence

Of the 400 participants selected for the Rasch analysis, one was excluded from person-level estimation because an extreme minimum score resulted in a non-finite standard error [36]. Principal component analysis of standardized residuals yielded an eigenvalue of 3.1 for the first residual contrast, suggesting the presence of residual structure beyond the primary Rasch dimension. However, disattenuated correlations involving the intermediate item cluster were greater than 0.70, exceeding the 0.57 threshold below which a contrast of this magnitude would suggest a substantively distinct secondary dimension. In addition, the essential unidimensionality ratio was 61.24%, exceeding the conventional 50% criterion [36]. Taken together, these findings were interpreted as supporting a dominant overarching dimension, while acknowledging some residual multidimensionality.

Yen’s Q3 residual correlations identified three locally dependent item pairs with correlations ≥ 0.70: t62–t63, t41–t42, and t31–t33. These associations were considered clinically interpretable because the paired items addressed closely related content [36, 38]. Because the objective was to evaluate the original 34-item instrument rather than to modify its content, all items were retained; however, the observed local dependence and its potential influence on parameter estimates and reliability were acknowledged when interpreting the Rasch findings [37].

### Model fit

Person and item fit statistics indicated that OUTFIT mean-square (MNSQ) values fell within the acceptable range for most items (Table 5). Given that OUTFIT is more sensitive to unexpected responses from low-ability persons on easy items, INFIT statistics were examined specifically for items exhibiting OUTFIT misfit, to determine whether the misfit also affected responses near the person’s ability level. Item 48 showed an INFIT MNSQ value of 1.27. Item 51 demonstrated misfit in INFIT statistics (MNSQ = 1.52; ZSTD = 5.74). Item 63 presented an INFIT MNSQ of 1.32, and item 64 showed an INFIT MNSQ of 1.35. Table 5 presents the OUTFIT fit statistics and corresponding item difficulty measures. Regarding person fit statistics, only 2.5% of patients (*n* = 10) exhibited OUTFIT ZSTD values greater than 3. No sociodemographic or clinical characteristics were identified that could explain a distinctive response pattern or a shared misfitting item.

**Table 5:** Item difficulty and OUTFIT fit statistics

Item	Difficulty	OUTFIT	
		MNSQ	ZSTD
t31	− 0.21	0.60	− 6.02
t32	0.02	0.67	− 4.40
t33	− 0.27	0.59	− 6.37
t34	0.56	0.88	− 1.08
t35	0.14	0.87	− 1.44
t36	0.18	1.03	0.40
t37	0.15	1.10	1.07
t38	0.07	0.87	− 1.59
t39	− 0.18	0.96	− 0.45
t40	− 0.59	1.29	3.78
t41	0.56	1.03	0.28
t42	0.47	0.88	− 1.16
t43	− 0.55	0.95	− 0.69
t44	− 0.29	1.06	0.80
t45	0.41	0.93	− 0.66
t46	− 0.06	0.73	− 3.61
t47	0.31	0.80	− 2.20
**t48**	**0.25**	**1.42**	**3.95**
t49	0.22	0.70	− 3.56
t50	− 0.24	0.95	− 0.62
**t51**	**0.50**	**1.43**	**3.64**
t52	0	0.99	− 0.07
t53	− 0.49	0.96	− 0.54
t54	− 0.50	0.80	− 2.99
t55	0.85	0.91	− 0.74
t56	− 0.20	1.14	1.72
t57	0.07	1.09	1.04
t58	− 1.16	1.22	2.84
t59	− 0.52	1.12	1.59
t60	− 0.95	1.13	1.77
t61	0.35	1.13	1.77
t62	0.36	1.24	2.26
**t63**	**0.22**	**1.47**	**4.48**
**t64**	**0.51**	**1.54**	**4.42**

Note: OUTFIT = outlier-sensitive fit statistic; MNSQ = mean square; ZSTD = standardized z-score. Items in bold indicate items with fit problems

#### DIF analysis

DIF was assessed by sex, educational level, employment status, age, and administration mode. Items 39 and 40 showed sex-related DIF (contrasts − 0.79 and − 0.82; *p* < 0.01), indicating greater endorsement of hair-related problems among women. Participants with lower educational attainment were more likely to endorse item 64 (contrast 0.70; *p* < 0.05), reflecting greater perceived lack of support from family or friends. No DIF meeting both the contrast and significance criteria was detected for employment status, age, or telephone versus face-to-face administration. Socioeconomic-status DIF could not be evaluated because comparison groups were too imbalanced (Supplementary Material 9).

### Rating scale functioning

“Not at all” was the most frequently endorsed category (48%), whereas “Very much” was the least frequent (10%). Average category measures and Andrich thresholds increased monotonically. Category INFIT and OUTFIT values were close to 1, and probability curves showed a distinct peak for each response option, providing no indication that categories should be collapsed [41] (Supplementary Material 10).

#### Person–item map

**Fig. 4:**
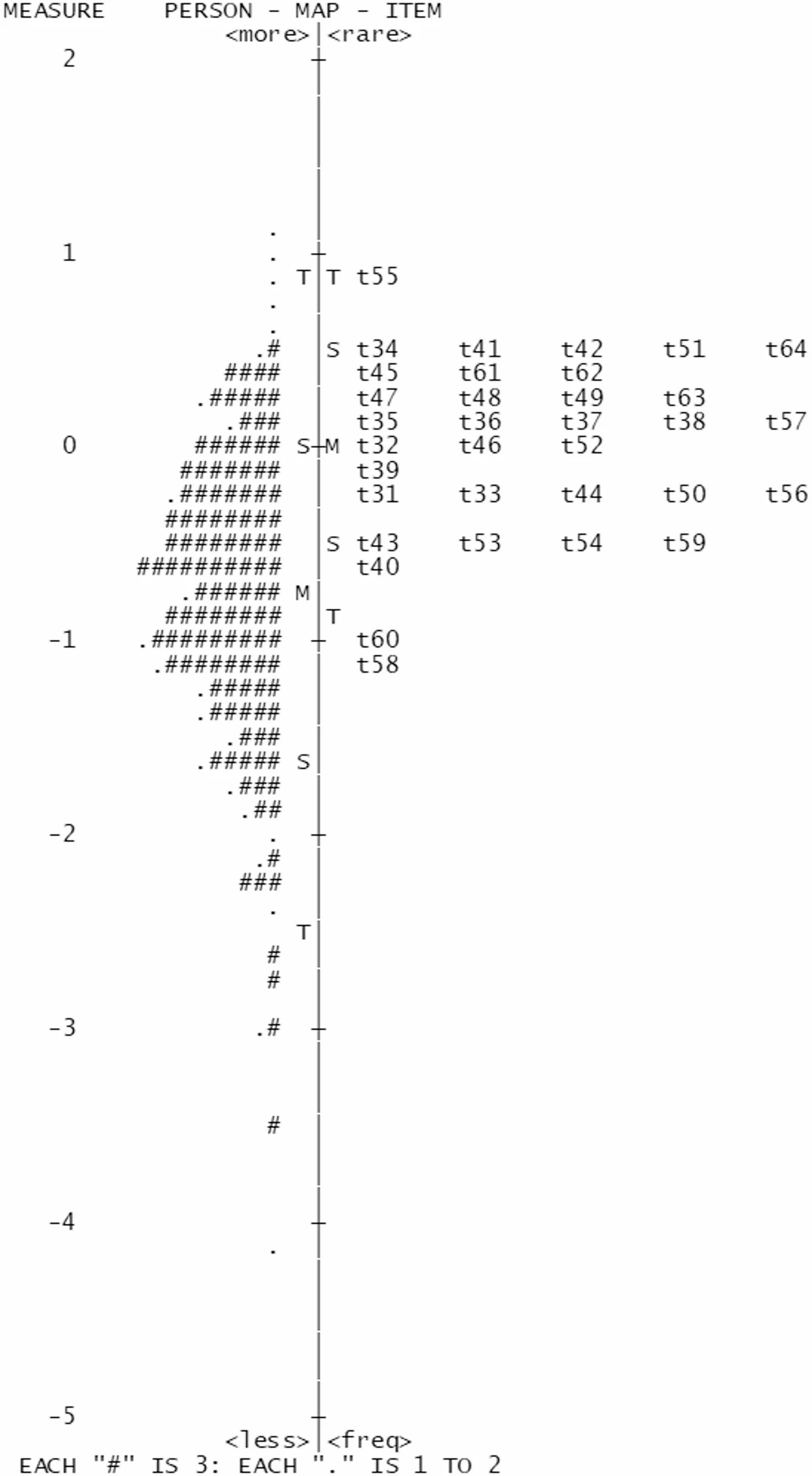
Wright map (person–item map) illustrating the relative ordering of items along the latent continuum, generated using Winsteps software

The Wright map placed item 55 as the most difficult and item 58 as the easiest to endorse (Fig. 4). The mean item location was slightly above the mean person location, indicating that items were somewhat more difficult than the impairment levels represented in this sample. Because the difference between means was < 1 logit, overall targeting was considered acceptable [36]. However, sparse item coverage at the lower end of the continuum suggests reduced measurement precision for respondents with minimal HRQoL impairment. This represents an item-targeting gap rather than a floor effect in observed domain scores. Several items overlapped in difficulty and content, particularly t41-t42 (swallowing), t36-t38 (voice), and t31-t33 (fatigue).

#### Reliability

Person reliability was 0.89 with a separation index of 2.92, while item reliability was 0.98, with a separation index of 6.82. These values suggest an adequate sample size for both persons and items, as well as strong discriminative capacity for the instrument. To evaluate the potential impact of local dependence on reliability estimates, an additional analysis was conducted by creating super-items [36], thereby eliminating residual correlations greater than 0.70. The results were consistent with the initial findings: person reliability was 0.88 (separation index = 2.76), and item reliability remained 0.98 (separation index = 6.70). These findings support the robustness of the original reliability estimates.

## Discussion

Split-sample factor analyses identified a four-domain model with substantially better fit than the original 17-scale structure, although RMSEA and SRMR remained above conventional thresholds. Partial attainment of conventional cutoffs can occur when long, multidimensional symptom modules are evaluated in a single culturally specific sample, particularly in the context of cross-cultural adaptation. The international field study of this module similarly reported a TLI of 0.94, below the value it had prespecified as indicating good fit, while concluding that the scale structure was supported [6]. These values therefore indicate approximate fit and do not invalidate the model, which reproduces the observed associations substantially better than the original structure. The empirical domains were used to generate study-specific scores for exploratory analyses. The moderate positive correlations among SI, OA, and CRC suggest that greater symptom burden, including oropharyngeal alterations, coexists with more cancer-related concerns. In contrast, the weak correlations between SO and the other domains suggest that perceived lack of social support represents a relatively distinct construct. These cross-sectional correlations do not imply directionality or causality. Correlations were strongest between SI and conceptually related QLQ-C30 scales, while CRC and OA showed mostly moderate associations in the expected directions. The weak correlations between SO and QLQ-C30 scales likely reflect the conceptual distinctness of social support, which is not directly represented in the core questionnaire.

The four-domain structure should be considered a preliminary, population-specific finding rather than a replacement for the original EORTC scoring system. Cultural or translation problems are an unlikely sole explanation for the poor fit of the original model because adaptation followed EORTC procedures and cognitive interviews identified no comprehension difficulties [9, 10]. The sample’s clinical homogeneity may have restricted variability in symptom burden and affected fit, but this explanation remains speculative. The official 17-scale structure should remain the standard for scoring and cross-study comparison until the alternative structure is replicated in independent, clinically diverse samples [6].

The distinct performance of the SO domain may partly reflect the recruitment context. Two of its three items assess support from healthcare professionals, and all participants were recruited at a national oncology referral center, where favorable perceptions of specialized care may be expected. Australian thyroid cancer survivors have similarly reported support from clinicians, family members, and social networks despite persistent unmet supportive care needs [42]. Family and community support patterns in Colombia may also have contributed to this finding; however, they were not directly assessed and this explanation remains hypothetical. Although item t64 showed DIF by educational level and internal consistency increased when it was omitted, these findings do not by themselves justify its removal. Item t64 is the only item assessing support from family or friends and therefore captures an important component of the construct that is not represented by the other two items. Its deletion would restrict the domain to professional support and leave two locally dependent items. We recommend further evaluation of t64 across educational groups and healthcare settings.

Regarding reliability, Cronbach’s alpha, McDonald’s omega, and greatest lower bound coefficients supported adequate internal consistency across the four identified domains. Deletion of most individual items produced little change in the alpha and omega coefficients of their respective domains, suggesting that most items contributed adequately to the internal consistency of the domains. However, removal of item t64 substantially increased the coefficients for the SO domain, indicating that the contribution of this item warrants further investigation. The Cronbach’s alpha obtained for the SO domain was similar to that reported for the corresponding scale in the international validation, whereas direct comparisons for the remaining domains were not possible because of the four-domain structure identified in the present study [6].

Test–retest concordance was good for SI, CRC, and OA and moderate for SO. In the test–retest subsample, baseline was completed face to face before the clinical consultation, whereas the retest was conducted by telephone. The comparatively lower SO coefficient may therefore partly reflect the change in administration mode or changes in perceived professional support following the consultation, although these potential influences could not be evaluated separately in the present study.

SI and CRC showed statistically significant changes after surgical reintervention, whereas OA and SO did not. All domains had descriptive changes of at least 4 points at one or more assessments, but statistical change alone does not establish clinically important change. Moreover, the analysis used no external anchor and included a selected subgroup undergoing reintervention. These findings should therefore be interpreted as preliminary evidence of longitudinal change rather than definitive responsiveness across all domains. Future prospective studies incorporating external anchors are needed to establish the minimal important difference for each domain.

Rasch findings also require balanced interpretation. The disattenuated correlations and essential-unidimensionality ratio supported a dominant dimension, but the first residual contrast exceeded the preferred threshold and three item pairs showed substantial local dependence [37]. Four items exceeded the OUTFIT criterion, although person misfit was uncommon and no common profile was identified [37]. Rating scale functioning was appropriate. Mean targeting was acceptable, but the lower-end item gap suggests less precision among patients with minimal impairment and potentially greater information among more symptomatic patients. Person and item reliability and separation were high and remained similar in the super-item analysis [43, 44].

At the item level, item t48 (shoulder mobility) showed low EFA loading and borderline OUTFIT misfit, consistent with its weaker performance in international validation [6]. It was retained given its clinical relevance (shoulder dysfunction is a recognized morbidity after lateral neck dissection) [45], and the adequate reliability and responsiveness of its parent SI domain. Item t51 (concerns about discontinuing thyroid hormone therapy for radioactive iodine treatment) showed low communality and borderline OUTFIT/INFIT misfit, possibly reflecting evolving treatment modalities such as injectable thyrotropin and reduced radioactive iodine use [46]. Item t64 improved internal consistency when removed from the SO domain, showed mixed Rasch misfit, and displayed DIF by education, with higher-attainment patients perceiving less lack of support, consistent with prior studies [47]. Items t39 and t40 showed sex-related DIF, consistent with literature showing hair loss affects women more than men [48]. Future studies should consider evaluating these items separately [36].

In Colombia, the THYCA-QoL questionnaire has also been validated for assessing HRQoL in patients with thyroid cancer. This instrument comprises 24 items but does not assess social aspects. It demonstrated lower internal consistency values for some domains and ICC values < 0.5 for most domains [49], in contrast to the findings of the present study, where the EORTC QLQ-THY34 demonstrated superior psychometric properties in terms of reliability and validity. However, these results should be interpreted cautiously because the two instruments were evaluated in separate studies rather than through a direct head-to-head comparison.

Among the study limitations, the research was conducted at a single healthcare institution, and a large proportion of participants originated from Bogotá, Cundinamarca, Tolima, and Boyacá. Nevertheless, most cultural regions of the country were represented, providing contextual heterogeneity. However, the sample was predominantly composed of female patients with papillary histology and excellent performance status; this may limit generalizability to patients with poorer functional status or more advanced disease. Known-groups validity was not evaluated because sufficiently large and clinically distinct comparison groups were not available. Future studies should test prespecified hypotheses across disease stage, treatment status, postoperative complications, and other clinically relevant groups. Furthermore, aside from a validation study conducted in China [50], few studies have explored alternative factorial structures, and additional research evaluating the clinimetric properties of this questionnaire is warranted. Finally, applying one Rasch model to all 34 items alongside a multidomain CTT solution requires replication and further methodological evaluation.

We encourage future validation studies of the EORTC QLQ-THY34 in other populations to examine whether comparable alternative factor structures emerge. Such studies would help determine whether the four-domain solution reflects a population-specific pattern or a potentially more generalizable refinement of the original module.

## Conclusion

In conclusion, analyses conducted using both Classical Test Theory and the Rasch model indicate that the Colombian Spanish adaptation of the EORTC QLQ-THY34 has psychometric evidence for assessing health-related quality of life in patients with thyroid cancer in Colombia. While the empirical four-domain structure should be considered preliminary, these findings support its implementation in clinical practice and research, with the understanding that the original EORTC scoring system should remain the standard for cross-study comparisons.

## Supplementary Information

Below is the link to the electronic supplementary material.

Supplementary Material

## Data Availability

The datasets generated and analyzed during the current study are stored in an institutional REDCap database and are not publicly available due to institutional data protection policies and the presence of sensitive clinical information. Data may be available from the corresponding author upon reasonable request and subject to institutional approval.
