# Supplementary material for: Validation for use in Colombia of the EORTC QLQ–THY34 questionnaire to measure quality of life in patients with thyroid cancer
Source: Qual Life Res. 2026 Sep 21;35(10):288. doi: 10.1007/s11136-026-04392-9 (PMC13593722; doi:10.1007/s11136-026-04392-9)
Supplement: Supplementary file 1 [file 11136_2026_4392_MOESM1_ESM.docx]

**Supplementary appendix**

**Supplementary Material 1:**

**Sample size calculations for psychometric analyses**

| **Psychometric property** | **Assumptions** | **Minimum sample size** | **Statistical software** | **Reference** |
| --- | --- | --- | --- | --- |
| Exploratory Factor Analysis (EFA) | Communalities  0.40–0.70; ≥3 items per factor | 250 |  | MacCallum et al., 1999 |
| Confirmatory Factor Analysis (CFA) | 5–10 participants per item; minimum 100–200 observations | 250 |  | Caycho-Rodríguez, 2018 |
| Construct validity (convergent)^a^ | r = 0.40 (H₁) vs 0.10 (H₀) | 78 | PASS® | Bonett & Wright, 2000 |
| Internal consistency ^a^ | α = 0.80 (H₁) vs 0.70 (H₀) | 96 | PASS® | Bonett, 2002 |
| Test–retest reliability ^a^ | Concordance correlation = 0.75 (H₁) vs 0.60 (H₀) | 81 | PASS® | Lin, 1989 |
| Responsiveness ^a^ | SRM = 0.20; 3 measurements; correlation = 0.10; ε = 1 | 42 | G*Power | Lubitz et al., 2017 |
| Rasch model analysis | Sample size based on EORTC guidelines for stable estimation | 400 |  | EORTC guidelines |
| ^a^ significance level of 0.05 and statistical power of 0.80. The final sample size of 500 participants exceeded the minimum requirements for all psychometric analyses. | | | | |

**Supplementary Material 2 Additional methodological details**

| **Analysis** | **Indicator** | **Criteria** | **Software/Package** |
| --- | --- | --- | --- |
| Factorability | Bartlett’s test | p < 0.05 | R (psych) |
|  | KMO | ≥ 0.70 | R (psych) |
| EFA | Factor loadings | ≥ 0.40 | R (psych) |
|  | Communalities | ≥ 0.40 | R (psych) |
| CFA (absolute fit) | χ²/df | < 5 | R (lavaan) |
|  | GFI | close to 1 | R (lavaan) |
|  | AGFI | ≥ 0.90 | R (lavaan) |
|  | RMSEA | ≤ 0.08 | R (lavaan) |
|  | RMR | close to 0 | R (lavaan) |
|  | SRMR | ≤ 0.08 | R (lavaan) |
| CFA (incremental fit) | CFI | ≥ 0.95 | R (lavaan) |
|  | TLI | ≥ 0.95 | R (lavaan) |
|  | NFI | ≥ 0.90 | R (lavaan) |
|  | IFI | ≥ 0.90 | R (lavaan) |
| Internal consistency | Cronbach’s alpha | 0.70–0.90 | R (psych) |
|  | McDonald’s omega | ≥ 0.70 | R (psych) |
|  | Greatest lower bound (GLB) | higher values indicate better reliability | R (psych) |
| Construct validity | Spearman correlation | p < 0.05 | R (stats) |
| Test–retest reliability | Lin’s concordance correlation coefficient (CCC) | <0.50 poor; 0.50–0.75 moderate; 0.75–0.90 good; >0.90 excellent | R (DescTools) |
| Responsiveness | Friedman test | p < 0.05 | R (stats) |
|  | Wilcoxon signed-rank test (Bonferroni corrected) | p < 0.05 | R (stats) |
| Rasch model | First residual contrast eigenvalue | < 2.0 preferred | Winsteps® |
|  | Disattenuated correlations | > 0.70 | Winsteps® |
|  | Essential unidimensionality | > 50% | Winsteps® |
|  | Yen’s Q3 residual correlations | < 0.70 | Winsteps® |
|  | Infit/Outfit MNSQ | 0.60–1.40 | Winsteps® |
|  | ZSTD | –2 to 2 | Winsteps® |
|  | DIF contrast | > 0.64 logits | Winsteps® |
|  | DIF significance | p < 0.05 | Winsteps® |
|  | Person/item reliability | close to 1 | Winsteps® |
|  | Separation index (persons) | >1.5 acceptable; >2.0 good; >3.0 excellent | Winsteps® |
|  | Separation index (items) | >1.5 acceptable; >2.5 good | Winsteps® |
|  | Category functioning | ordered thresholds and distinct category peaks | Winsteps® |

**Supplementary Material 3. Participant recruitment and analysis flow**

**Supplementary Material 4 -Paralel analysis and scree plot**

**Supplementary Material 5**

**Item stems and their mapping to original EORTC scales and empirical domains**

| **Item** | **Item stem** | **Original EORTC scale** | **Empirical domain** |
| --- | --- | --- | --- |
| t31 | Sudden attacks of tiredness | Exhaustion (EX) | SI |
| t32 | Mentally exhausted |  |  |
| t33 | Physically exhausted |  |  |
| t39 | Thin or lifeless hair | Hair Problems (HA) |  |
| t40 | Lost hair |  |  |
| t43 | Dry mouth | Dry Mouth (DM) |  |
| t44 | Problems tolerating heat or cold | Altered Temperature Tolerance (TO) |  |
| t46 | Felt restless or agitated | Treatment- and Disease-related Worry (TD) |  |
| t47 | Rapid heartbeat | Rapid Heartbeat (RH) |  |
| t48 | Problems raising/moving arm | Shoulder Functioning (SH) |  |
| t53 | Pain in joints | Joint Pain (JP) |  |
| t54 | Tingling/numbness, fingers or toes | Tingling or Numbness (TI) |  |
| t55 | Tingling/numbness around mouth |  |  |
| t56 | Muscle cramps | Cramps (CR) |  |
| t61 | Problems with job or education | Impact on Job or Education (JE) |  |
| t62 | Supported by doctors | Lacking Social Support (SO) | SO |
| t63 | Supported by other health professionals |  |  |
| t64 | Supported by family or friends |  |  |
| t45 | Felt less physically attractive | Body Image (BI) | CRC |
| t50 | Worried about recurrence | Treatment- and Disease-related Worry (TD) |  |
| t51 | Worried about stopping hormone tablets |  |  |
| t52 | Worried about lifelong medication |  |  |
| t57 | Upsetting to see close ones distressed | Worry about Important Others (WO) |  |
| t58 | Worried about future of important others |  |  |
| t59 | Worried how close ones will cope |  |  |
| t60 | Worried about being a burden |  |  |
| t34 | Pain in throat | Discomfort in the Head and Neck (DI) | OA |
| t35 | Discomfort in neck |  |  |
| t36 | Hoarseness | Voice (VO) |  |
| t37 | Voice sounded different |  |  |
| t38 | Tired voice |  |  |
| t41 | Problems swallowing solid food | Swallowing (SW) |  |
| t42 | Choked when swallowing |  |  |
| t49 | Knot in throat | Discomfort in the Head and Neck (DI) |  |

*SI: Symptoms and Impact; SO: Lack of Social Support; CRC: Cancer-Related Concerns; OA: Oropharyngeal Alterations.* Item stems are presented as brief thematic descriptors and do not reproduce the verbatim wording or response format of the original EORTC QLQ-THY34 items, which remain the intellectual property of the EORTC Quality of Life Group. Full item wording is available upon request or license from EORTC.

**Distribution of Factor Loadings Across Domains Derived from the Exploratory Factor Analysis**

| **Item** | **SI** | **OA** | **CRC** | **SO** | **Uniqueness** |
| --- | --- | --- | --- | --- | --- |
| t31 | 0.82 |  |  |  | 0.321 |
| t32 | 0.84 |  |  |  | 0.285 |
| t33 | 0.80 |  |  |  | 0.313 |
| t39 | 0.61 |  |  |  | 0.500 |
| t40 | 0.53 |  |  |  | 0.561 |
| t43 | 0.41 |  |  |  | 0.736 |
| t44 | 0.59 |  |  |  | 0.612 |
| t46 | 0.66 |  |  |  | 0.525 |
| t47 | 0.59 |  |  |  | 0.636 |
| t48 | 0.36 |  |  |  | 0.830 |
| t53 | 0.48 |  |  |  | 0.727 |
| t54 | 0.59 |  |  |  | 0.582 |
| t55 | 0.49 |  |  |  | 0.676 |
| t56 | 0.44 |  |  |  | 0.725 |
| t61 | 0.49 |  |  |  | 0.661 |
| t62 |  |  |  | 0.96 | 0.082 |
| t63 |  |  |  | 0.86 | 0.227 |
| t64 |  |  |  | 0.55 | 0.598 |
| t45 |  |  | 0.41 |  | 0.723 |
| t50 | 0.54 |  | 0.34 |  | 0.588 |
| t51 |  |  | 0.50 |  | 0.714 |
| t52 | 0.46 |  | 0.44 |  | 0.569 |
| t57 |  |  | 0.75 |  | 0.419 |
| t58 |  |  | 0.72 |  | 0.442 |
| t59 |  |  | 0.83 |  | 0.306 |
| t60 |  |  | 0.58 |  | 0.576 |
| t34 |  | 0.55 |  |  | 0.515 |
| t35 |  | 0.59 |  |  | 0.576 |
| t36 |  | 0.77 |  |  | 0.306 |
| t37 |  | 0.75 |  |  | 0.291 |
| t38 |  | 0.77 |  |  | 0.371 |
| t41 |  | 0.70 |  |  | 0.392 |
| t42 |  | 0.66 |  |  | 0.471 |
| t49 |  | 0.57 |  |  | 0.507 |
| SI: Symptoms and Impact; SO: Lack of Social Support; CRC: Cancer-Related Concerns; and OA: Oropharyngeal Alterations. | | | | | |

**Supplementary Material 6**

**Scoring algorithm used to conduct analyses of construct validity, reliability, and responsiveness**

**Symptoms and impact - SI**

si <- base %>%

select(c(t31 , t32, t33, t39, t40, t43, t44, t46, t47, t48, t53, t54, t55, t56, t61))

rs_si <- (rowSums(si)/15)

s_si <- ((rs_si - 1)/3)*100

**Lack of ocial Support - SO**

so <- base %>%

select(c(t62, t63, t64))

rs_so <- (rowSums(so)/3)

s_so <- ((rs_so-1)/3)*100

Items 62, 63 and 64 where reverse

**Cancer-Related Concerns - CRC**

crc <- base %>%

select(c(t45, t50, t51, t52, t57, t58, t59, t60))

rs_crc <- (rowSums(crc)/8)

s_crc <- ((rs_crc-1)/3)*100

**Oropharyngeal Alterations**

aof <- base%>%

select(c(t35, t36, t37, t38, t41, t42, t34, t49))

rs_aof <- (rowSums(aof)/8)

s_aof<- ((rs_aof - 1)/3)*100

**Supplementary Material 7**

**Cronbach’s Alpha and McDonald’s Omega for the Overall Questionnaire and by Domain**

| **Domain** | **Cronbach´s Alpha** | **McDonald Omega** **(ω)** |
| --- | --- | --- |
| **Overall questionnaire** | 0.91 | 0.93 |
| **SI** | 0.91 | 0.90 |
| **SO** | 0.86 | 0.84 |
| **CRC** | 0.84 | 0.82 |
| **OA** | 0.87 | 0.89 |
| SI: Symptoms and Impact; SO: Lack of Social Support; CRC: Cancer-Related Concerns; and OA: Oropharyngeal Alterations. | | |

**Cronbach’s Alpha, McDonald’s Omega, and Greatest Lower Bound (GLB) Coefficients for the Overall Questionnaire and After Item-by-Item Deletion**

| **Item deletion** | **Cronbach´s Alpha** | **McDonald Omega** **(ω)** | **GLB** |
| --- | --- | --- | --- |
| Cuestionario completo | 0.91 | 0.93 | 0.99 |
| t31 | 0.90 | 0.89 | 0.99 |
| t32 | 0.90 | 0.89 | 0.99 |
| t33 | 0.90 | 0.89 | 0.99 |
| t34 | 0.91 | 0.90 | 0.99 |
| t35 | 0.91 | 0.90 | 0.99 |
| t36 | 0.91 | 0.90 | 0.99 |
| t37 | 0.91 | 0.90 | 0.99 |
| t38 | 0.91 | 0.90 | 0.99 |
| t39 | 0.91 | 0.89 | 0.99 |
| t40 | 0.91 | 0.90 | 0.99 |
| t41 | 0.91 | 0.90 | 0.99 |
| t42 | 0.91 | 0.90 | 0.99 |
| t43 | 0.91 | 0.90 | 0.99 |
| t44 | 0.91 | 0.90 | 0.99 |
| t45 | 0.91 | 0.90 | 0.99 |
| t46 | 0.91 | 0.90 | 0.99 |
| t47 | 0.91 | 0.90 | 0.99 |
| t48 | 0.91 | 0.90 | 0.99 |
| t49 | 0.91 | 0.90 | 0.99 |
| t50 | 0.91 | 0.90 | 0.99 |
| t51 | 0.91 | 0.90 | 0.99 |
| t52 | 0.91 | 0.90 | 0.99 |
| t53 | 0.90 | 0.90 | 0.99 |
| t54 | 0.91 | 0.90 | 0.99 |
| t55 | 0.91 | 0.90 | 0.99 |
| t56 | 0.91 | 0.90 | 0.99 |
| t57 | 0.91 | 0.90 | 0.99 |
| t58 | 0.91 | 0.90 | 0.99 |
| t59 | 0.91 | 0.90 | 0.99 |
| t60 | 0.91 | 0.90 | 0.99 |
| t61 | 0.91 | 0.90 | 0.99 |
| t62 | 0.91 | 0.90 | 0.99 |
| t63 | 0.91 | 0.90 | 0.99 |
| t64 | 0.91 | 0.90 | 0.99 |

**Cronbach’s Alpha and McDonald’s Omega Coefficients After Item-by-Item Deletion for Each Domain**

| **Item deleted** | **SI ^a^** | **SI^b^** | **SO ^a^** | **SO ^b^** | **CRC ^a^** | **CRC ^b^** | **OA ^a^** | **OA ^b^** |
| --- | --- | --- | --- | --- | --- | --- | --- | --- |
| t31 | 0,90 | 0,89 | 0,86 | 0,84 | 0,84 | 0,82 | 0,87 | 0,89 |
| t32 | 0,90 | 0,89 | 0,86 | 0,84 | 0,84 | 0,82 | 0,87 | 0,89 |
| t33 | 0,90 | 0,89 | 0,86 | 0,84 | 0,84 | 0,82 | 0,87 | 0,89 |
| t34 | 0,91 | 0,90 | 0,86 | 0,84 | 0,84 | 0,82 | 0,86 | 0,88 |
| t35 | 0,91 | 0,90 | 0,86 | 0,84 | 0,84 | 0,82 | 0,85 | 0,88 |
| t36 | 0,91 | 0,90 | 0,86 | 0,84 | 0,84 | 0,82 | 0,85 | 0,88 |
| t37 | 0,91 | 0,90 | 0,86 | 0,84 | 0,84 | 0,82 | 0,84 | 0,85 |
| t38 | 0,91 | 0,90 | 0,86 | 0,84 | 0,84 | 0,82 | 0,84 | 0,85 |
| t39 | 0,91 | 0,89 | 0,86 | 0,84 | 0,84 | 0,82 | 0,87 | 0,89 |
| t40 | 0,91 | 0,90 | 0,86 | 0,84 | 0,84 | 0,82 | 0,87 | 0,89 |
| t41 | 0,91 | 0,90 | 0,86 | 0,84 | 0,84 | 0,82 | 0,85 | 0,85 |
| t42 | 0,91 | 0,90 | 0,86 | 0,84 | 0,84 | 0,82 | 0,85 | 0,85 |
| t43 | 0,91 | 0,90 | 0,86 | 0,84 | 0,84 | 0,82 | 0,87 | 0,89 |
| t44 | 0,91 | 0,90 | 0,86 | 0,84 | 0,84 | 0,82 | 0,87 | 0,89 |
| t45 | 0,91 | 0,90 | 0,86 | 0,84 | 0,83 | 0,81 | 0,87 | 0,89 |
| t46 | 0,91 | 0,90 | 0,86 | 0,84 | 0,84 | 0,82 | 0,87 | 0,89 |
| t47 | 0,91 | 0,90 | 0,86 | 0,84 | 0,84 | 0,82 | 0,87 | 0,89 |
| t48 | 0,91 | 0,90 | 0,86 | 0,84 | 0,84 | 0,82 | 0,87 | 0,89 |
| t49 | 0,91 | 0,90 | 0,86 | 0,84 | 0,84 | 0,82 | 0,85 | 0,88 |
| t50 | 0,91 | 0,90 | 0,86 | 0,84 | 0,82 | 0,80 | 0,87 | 0,89 |
| t51 | 0,91 | 0,90 | 0,86 | 0,84 | 0,83 | 0,81 | 0,87 | 0,89 |
| t52 | 0,91 | 0,90 | 0,86 | 0,84 | 0,83 | 0,80 | 0,87 | 0,89 |
| t53 | 0,90 | 0,90 | 0,86 | 0,84 | 0,84 | 0,82 | 0,87 | 0,89 |
| t54 | 0,91 | 0,90 | 0,86 | 0,84 | 0,84 | 0,82 | 0,87 | 0,89 |
| t55 | 0,91 | 0,90 | 0,86 | 0,84 | 0,84 | 0,82 | 0,87 | 0,89 |
| t56 | 0,91 | 0,90 | 0,86 | 0,84 | 0,84 | 0,82 | 0,87 | 0,89 |
| t57 | 0,91 | 0,90 | 0,86 | 0,84 | 0,81 | 0,78 | 0,87 | 0,89 |
| t58 | 0,91 | 0,90 | 0,86 | 0,84 | 0,83 | 0,79 | 0,87 | 0,89 |
| t59 | 0,91 | 0,90 | 0,86 | 0,84 | 0,81 | 0,76 | 0,87 | 0,89 |
| t60 | 0,91 | 0,90 | 0,86 | 0,84 | 0,82 | 0,80 | 0,87 | 0,89 |
| t61 | 0,91 | 0,90 | 0,86 | 0,84 | 0,84 | 0,82 | 0,87 | 0,89 |
| t62 | 0,91 | 0,90 | 0,76 | 0,68 | 0,84 | 0,82 | 0,87 | 0,89 |
| t63 | 0,91 | 0,90 | 0,72 | 0,66 | 0,84 | 0,82 | 0,87 | 0,89 |
| t64 | 0,91 | 0,90 | **0,93** | **0,90** | 0,84 | 0,82 | 0,87 | 0,89 |
| ^a:^ Cronbach’s Alpha^; b:^ McDonald’s Omega | | | | | | | | |

**Supplementary Material 8**

**Spearman Correlations Between the Domains of the EORTC QLQ-C30 Core Questionnaire and the Domains Derived from the Confirmatory Factor Analysis.**

| **Escale/Domain** | **SEI** | **FAS** | **PRC** | **AOF** |
| --- | --- | --- | --- | --- |
| **QL2** | -0.646 ^c^ | -0.214 ^c^ | -0.453 ^c^ | -0.409 ^c^ |
| **PF2** | -0.585 ^c^ | -0.070^a^ | -0.380 ^c^ | -0.477 ^c^ |
| **RF2** | -0.595 ^c^ | -0.074 ^a^ | -0.391 ^c^ | -0.411 ^c^ |
| **EF** | -0.633 ^c^ | -0.106^a^ | -0.466 ^c^ | -0.395 ^c^ |
| **CF** | -0.618 ^c^ | -0.061^a^ | -0.332 ^c^ | -0.389 ^c^ |
| **SF** | -0.487 ^c^ | -0.068^a^ | -0.446 ^c^ | -0.394 ^c^ |
| **FA** | 0.750 ^c^ | 0.110^a^ | 0.422 ^c^ | 0.485 ^c^ |
| **NV** | 0.310 ^c^ | 0.016^a^ | 0.139^b^ | 0.202 ^c^ |
| **PA** | 0.656 ^c^ | 0.053^a^ | 0.360 ^c^ | 0.478 ^c^ |
| **DY** | 0.384 ^c^ | 0.140^b^ | 0.310^b^ | 0.412 ^c^ |
| **SL** | 0.551 ^c^ | 0.034^a^ | 0.354 ^c^ | 0.429 ^c^ |
| **AP** | 0.448 ^c^ | 0.029^a^ | 0.258 ^c^ | 0.326 ^c^ |
| **CO** | 0.368 ^c^ | -0.041^a^ | 0.188 ^c^ | 0.241 ^c^ |
| **DI** | 0.189 ^c^ | 0.047^a^ | 0.143 ^c^ | 0.200 ^c^ |
| **FI** | 0.336 ^c^ | 0.056^a^ | 0.378 ^c^ | 0.302 ^c^ |
| a: p>0.05; b: p< 0.05; c: p< 0.01; QL2.Global health status; PF: Physical functioning; RF: Role functioning; EF: Emotional functioining; CF: Cognitive functioning; SF: Social functioning; FA: Fatigue; NV: Nausea and vomiting; PA: Pain; DY: Dysnea; SL: Insomnia; AP: Appetite loss; CO: Constipation; DI: Diarrhoea; FI: Financial difficulties; SI: Symptoms and Impact; SO: Lack of Social Support; CRC: Cancer-Related Concerns; and OA: Oropharyngeal Alterations. | | | | |

**Supplementary Material 9**

**Differential Item Functioning (DIF) Analysis by Sociodemographic Characteristics**

|  | **Sex** | | | **Age** | | | **Employment Status** | | | **Education** | | |
| --- | --- | --- | --- | --- | --- | --- | --- | --- | --- | --- | --- | --- |
| **Ítem** | **DIF contrast** | **Mantel statistic** | **P** | **DIF contrast** | **Mantel**  **Statistic** | **P** | **DIF contrast** | **Mantel statistic** | **P** | **DIF contrast** | **Mantel statistic** | **P** |
| 31 | -0,32 | 11,87 | 0,28 | 0 | 0,71 | 0,40 | 0,11 | 0,22 | 0,64 | 0,06 | 0,04 | 0,85 |
| 32 | -0,19 | 0,03 | 0,86 | -0,2 | 30,83 | 0,08 | 0,3 | 73,42 | 0,01 | 0,19 | 0,79 | 0,37 |
| 33 | 0 | 0,17 | 0,68 | -0,03 | 0,06 | 0,80 | 0,03 | 0,00 | 0,99 | -0,03 | 0,26 | 0,61 |
| 34 | 0 | 12,09 | 0,27 | 0 | 15,63 | 0,21 | -0,15 | 3,29 | 0,07 | 0,3 | 3,03 | 0,08 |
| 35 | 0,51 | 99,12 | 0,00 | 0,11 | 0,51 | 0,47 | -0,05 | 0,28 | 0,60 | 0,3 | 1,83 | 0,18 |
| 36 | 0,11 | 0,16 | 0,69 | 0,27 | 30,99 | 0,08 | -0,39 | 8,02 | 0,00 | -0,04 | 0,00 | 0,99 |
| 37 | 0,39 | 24,03 | 0,12 | 0,32 | 34,17 | 0,06 | -0,34 | 75,88 | 0,01 | -0,14 | 0,34 | 0,56 |
| 38 | 0,22 | 0,83 | 0,36 | 0,05 | 0,52 | 0,47 | 0,07 | 0,03 | 0,87 | -0,02 | 0,05 | 0,83 |
| **39** | **-0,79** | **70,63** | **0,01** | -0,21 | 0,47 | 0,49 | 0,31 | 43,66 | 0,04 | -0,34 | 4,14 | 0,04 |
| **40** | **-0,82** | **126,46** | **0,00** | -0,38 | 39,43 | 0,05 | 0,33 | 43,49 | 0,04 | -0,33 | 2,46 | 0,12 |
| 41 | 0,11 | 0,00 | 1,00 | 0,48 | 6,53 | 0,01 | -0,5 | 100,08 | 0,00 | 0,11 | 0,01 | 0,90 |
| 42 | 0,22 | 0,34 | 0,56 | 0,56 | 103,64 | 0,00 | -0,54 | 141,39 | 0,00 | 0,06 | 0,15 | 0,70 |
| 43 | -0,1 | 0,33 | 0,56 | 0,3 | 4,86 | 0,03 | -0,14 | 13,53 | 0,24 | 0,2 | 1,74 | 0,19 |
| 44 | 0,05 | 0,01 | 0,93 | 0,19 | 17,10 | 0,19 | 0,14 | 19,53 | 0,16 | 0,12 | 0,09 | 0,77 |
| 45 | 0 | 0,69 | 0,41 | -0,06 | 0,15 | 0,70 | 0 | 0,05 | 0,82 | 0 | 0,26 | 0,61 |
| 46 | -0,14 | 0,37 | 0,54 | 0,21 | 22,65 | 0,13 | -0,14 | 15,90 | 0,21 | 0,03 | 0,35 | 0,55 |
| 47 | -0,31 | 28,87 | 0,09 | 0 | 0,26 | 0,61 | 0,02 | 0,07 | 0,80 | -0,03 | 0,02 | 0,89 |
| 48 | -0,02 | 0,04 | 0,84 | 0,07 | 0,39 | 0,53 | -0,17 | 36,55 | 0,06 | 0,25 | 1,81 | 0,18 |
| 49 | 0,5 | 122,23 | 0,00 | -0,21 | 10,65 | 0,30 | 0,11 | 0,40 | 0,53 | -0,35 | 3,26 | 0,07 |
| 50 | 0,11 | 0,31 | 0,58 | -0,33 | 61,17 | 0,01 | 0,21 | 55,99 | 0,02 | -0,27 | 1,62 | 0,20 |
| 51 | -0,35 | 21,67 | 0,14 | -0,45 | 74,56 | 0,01 | 0,18 | 33,76 | 0,07 | -0,02 | 0,01 | 0,94 |
| 52 | 0,1 | 20,36 | 0,15 | -0,37 | 28,40 | 0,09 | 0,06 | 0,32 | 0,57 | -0,03 | 0,12 | 0,73 |
| 53 | -0,37 | 40,68 | 0,04 | 0,04 | 11,31 | 0,29 | -0,06 | 14,16 | 0,23 | 0,18 | 2,95 | 0,09 |
| 54 | 0 | 0,19 | 0,66 | -0,1 | 0,46 | 0,50 | 0,18 | 1,59 | 0,21 | -0,2 | 0,61 | 0,43 |
| 55 | -0,37 | 12,86 | 0,26 | -0,15 | 0,07 | 0,79 | 0,03 | 0,17 | 0,68 | -0,05 | 0,15 | 0,70 |
| 56 | -0,14 | 18,68 | 0,17 | 0,26 | 29,57 | 0,09 | -0,08 | 12,62 | 0,26 | -0,26 | 2,22 | 0,14 |
| 57 | 0,29 | 33,17 | 0,07 | 0,1 | 0,08 | 0,77 | -0,3 | 0,64 | 0,42 | -0,13 | 0,25 | 0,62 |
| 58 | 0,34 | 0,11 | 0,74 | 0 | 0,10 | 0,75 | 0 | 0,33 | 0,56 | 0 | 0,00 | 0,98 |
| 59 | 0,2 | 0,56 | 0,46 | -0,05 | 0,32 | 0,57 | -0,07 | 0,00 | 0,96 | -0,15 | 0,13 | 0,72 |
| 60 | -0,47 | 59,34 | 0,01 | -0,09 | 0,07 | 0,79 | -0,12 | 14,05 | 0,24 | 0,09 | 1,53 | 0,22 |
| 61 | 0,54 | 76,37 | 0,01 | -0,27 | 0,93 | 0,34 | 0,36 | 49,00 | 0,03 | 0,18 | 0,43 | 0,51 |
| 62 | 0,17 | 0,03 | 0,87 | -0,19 | 47,62 | 0,03 | 0,08 | 0,84 | 0,36 | 0 | 0,27 | 0,61 |
| 63 | 0,27 | 0,21 | 0,64 | -0,15 | 38,86 | 0,05 | 0 | 0,52 | 0,47 | -0,21 | 1,46 | 0,23 |
| 64 | 0,12 | 0,21 | 0,65 | 0,06 | 0,00 | 0,98 | 0,18 | 24,56 | 0,12 | **0,7** | **4,34** | **0,04** |

**Differential Item Functioning (DIF) Analysis according to the mode of questionnaire administration (telephone vs. face-to-face)**

|  | telephone vs. face-to-face | | |
| --- | --- | --- | --- |
| **Ítem** | **DIF contrast** | **Mantel statistic** | **P** |
| 31 | 0.06 | 0.04 | 0.83 |
| 32 | 0.16 | 1.35 | 0.24 |
| 33 | 0.07 | 0.26 | 0.60 |
| 34 | -0.18 | 0.78 | 0.37 |
| 35 | 0.06 | 0.60 | 0.43 |
| 36 | 0.00 | 0.53 | 0.46 |
| 37 | -0.19 | 2.31 | 0.12 |
| 38 | -0.21 | 1.35 | 0.24 |
| 39 | 0.22 | 1.55 | 0.21 |
| 40 | 0.33 | 5.41 | 0.02 |
| 41 | 0.14 | 1.08 | 0.29 |
| 42 | 0.14 | 2.85 | 0.09 |
| 43 | 0.17 | 0.46 | 0.49 |
| 44 | -0.14 | 2.56 | 0.10 |
| 45 | -0.07 | 0.99 | 0.31 |
| 46 | 0.08 | 0.52 | 0.46 |
| 47 | 0.00 | 0.00 | 0.97 |
| 48 | 0.12 | 0.97 | 0.32 |
| 49 | -0.05 | 0.07 | 0.78 |
| 50 | -0.28 | 4.57 | 0.03 |
| 51 | 0.11 | 4.12 | 0.04 |
| 52 | -0.08 | 0.29 | 0.58 |
| 53 | 0.06 | 0.03 | 0.85 |
| 54 | 0.14 | 3.01 | 0.08 |
| 55 | -0.28 | .10 | 0.29 |
| 56 | 0.12 | 1.23 | 0.26 |
| 57 | -0.07 | 0.24 | 0.62 |
| 58 | -0.07 | 0.18 | 0.66 |
| 59 | -0.15 | 0.47 | 0.49 |
| 60 | -0.03 | 0.35 | 0.55 |
| 61 | 0.00 | 0.56 | 0.45 |
| 62 | -0.07 | 0.87 | 0.34 |
| 63 | -0.18 | 2.72 | 0.09 |
| 64 | -0.18 | 2.77 | 0.09 |

**Supplementary Material 10**

**Category Probability Curves for the Four Response Categories of the Rating Scale.**
